# Prevalence of hepatitis B virus infection among blood donors at the Tamale Teaching Hospital, Ghana (2009)

**DOI:** 10.1186/1756-0500-5-115

**Published:** 2012-02-22

**Authors:** Julius Tieroyaare Dongdem, Sylvanus Kampo, Ireneous N Soyiri, Patrick Nsobila Asebga, Juventus B Ziem, Kenneth Sagoe

**Affiliations:** 1 Department of Medical Biochemistry, School of Medicine and Health Sciences, University for Development Studies, P. O. Box 1350, Tamale, Northern Region, Ghana; 2 Department of Allied Health Science, School of Medicine and Health Sciences, University for Development Studies, Tamale, Ghana; 3 Tamale Teaching Hospital, Ministry of Health, Tamale, Northern Region, Ghana; 4 School of Public Health, University of Ghana, Legon, Accra, Ghana; 5 Global Public Health, JCSMHS, MONASH University, 46150 Bandar Sunway, Selanger D.E, Malaysia; 6 Department of Microbiology. School of Medicine and Health Sciences, University for Development Studies, Tamale, Ghana

## Abstract

**Background:**

Despite education and availability of drugs and vaccines, hepatitis B virus (HBV) is still the most common severe liver infection in the world accounting for >1 million annual deaths worldwide. Transfusion of infected blood, unprotected sex and mother to child transmission are 3 key transmission routes of HBV in Ghana. There is high incidence of blood demanding health situations in northern Ghana resulting from anemia, accidents, malnutrition, etc. The higher the demand, the higher the possibility of transmitting HBV through infected blood. The aim of the investigation was to estimate the prevalence of HBV in blood donors which will provide justification for interventions that will help minimize or eliminate HBV infection in Ghana.

**Findings:**

We investigated the prevalence of HBV infection among blood donors at Tamale Teaching Hospital. The Wondfo HBsAg test kit was used to determine the concentration of HBsAg in 6,462 (576 voluntary and 5,878 replacement) donors as being ≥1 ng/ml. 10.79% of voluntary donors and 11.59% of replacement donors were HBsAg+. The 20-29 year group of voluntary donors was >2 times more likely to be HBsAg + than 40-60. Also the 20-29 year category of replacement donors was >4 times as likely to be HBsAg + than 50-69.

**Conclusions:**

Risk of infection was age, sex and donor type dependent. The 20-29 year category had the highest prevalence of HBsAg + cases, mostly males residing within the metropolis.

## Introduction

Hepatitis B virus (HBV) infection is associated with a wide variety of clinical manifestations including jaundice, hepatomegaly, anorexia, abdominal and gastric discomfort, etc. [1,2]. First discovered in 1967, HBV of the *Hapadnaviridae *family is a human blood-borne virus which is strictly hepatotropic [3-5]. HBV genotypes [6,7] have a distinct geographical distribution with genotypes A and D predominant in Europe, Middle East, Central Asia, Siberia and America. Genotypes B and C are predominant in East Asia while genotype E is more predominant in Africa [8]. Genotype F has been reported in Central America. Also, genotype G has been reported in the United States and France [9].

Hepatitis B is a major cause of liver disease worldwide [10]. Despite education, campaigns and availability of effective drugs e.g. interferon alpha, lamivudine, adefovir [11] and vaccines [12], the disease is still a major global health problem. There is serologic evidence of past or present HBV infection of approximately 2 billion people [13,14].

Hepatitis B is a contagious disease. Transfusion - transmitted HBV infection has become a major mode of transmission of HBV in high prevalence areas in sub Saharan Africa [15]. Transfusion of infected blood and unprotected sex are two key transmission routes of HBV in Ghana. Indeed, the WHO has documented that between 5 and 10% of HIV infections worldwide are transmitted through the transfusion of contaminated blood and blood products [16]. Thus, indicating that many more recipients of blood products are infected by HBV, HCV, syphilis and many other infectious agents [16]. The WHO has estimated that ≤50% of blood supply in sub-Saharan Africa is screened for HBsAg due to lack of perceived utility and/or lack of funds [17].

Blood safety therefore remains an issue of major concern in transfusion medicine in Sub Saharan Africa [15]. There is high incidence of blood-demanding health situations in northern Ghana resulting from anemia, malnutrition, accidents, surgical and obstetrical emergencies associated with blood loss, etc. The higher the demand for blood transfusion services, the higher the possibility of transmitting HBV and other blood-borne pathogens through contaminated blood. Illiteracy, lack of adequate health information, low socioeconomic situation in the region, inadequate trained health personnel and some inappropriate cultural practices linked with transmission dynamics including polygamy tend to augment the disease transmission. Furthermore, the National blood transfusion service's agenda, Government policies as well as that of championing stake holders to combat the disease are not clearly known. The study therefore was conducted to determine the prevalence of hepatitis B infection among blood donors at Tamale Teaching Hospital Blood Bank which will indicate the risk of transfusing infected blood if the blood is not properly screened. The investigation will provide justification for re-strategizing and initiating more sensitive interventions that will help minimize or possibly eliminate hepatitis B infection in Ghana.

## Methods

### Study population

The study samples included all individuals who donated blood from 01 January to 31 December, 2009. The study recruited 6,462 individuals in two categories; 5,878 blood replacement donors (individuals called upon to donate blood to patients) and 584 voluntary donors (individuals who willingly donated blood to the blood bank). Donor consent was obtained and confidentiality guaranteed. Clearance was also obtained from the Regional Health Directorate to use the data for the purpose of research only.

### Collection and preparation of specimen

A 5^cc ^syringe and needle were used to bleed approximately 5 ml of blood from each donor, transferred into a vacutainer and centrifuged at 1500 rpm for 3 min to obtain serum. The Wondfo One Step Cassette Style HBsAg test kit (Guangzhou Wondfo Biotech Co. Ltd, China) was used to determine the concentration of HBsAg as being ≥1 ng/ml. A test strip was immersed in each serum. The strips were removed after 10 sec and placed on a dry clean non-absorbent table surface for 15 min after which they were visualized.

### Interpretation of results

**Negative test for HBV: **Only one colored band appears on the control (C) region. **Positive test for HBV: **In addition to a pink colored control (C) band, a distinct pink colored band also appears in the test (T) region. **Invalid test for HBV: **A total absence of color in both regions. Also, results of recruits who could not tell their ages were eliminated.

### Analysis of data

The data obtained was double entered into a spread sheet. Descriptive analysis involved a display of summary statistics and cross tabulation of HBV, age groups and gender. We generated a binary logistic variable (as our primary dependent variable) to represent the status of HBV infection coded 0/1. The prevalence was then estimated as the proportion with infection alongside the exact binomial confidence interval. The statistical differences within age groups and between genders were tested with Pearson's chi square as well as bivariate logistic regression. A logistic regression was further used to identify the independent associations between the categories and the respective probability of HBV infection among individuals at the time of blood donation.

The data was analyzed using Stata version 11.2 (Special Edition, College Station, Texas 77845 USA) statistical package set at 95% Confidence Interval (and a *p*-value < 0.05 as considered statistically significant).

## Results

### Voluntary blood donors and HBV test results

Out of 584 voluntary donors, 2.05% were female. The remaining 97.95% were male. Test results of 8 voluntary donors were excluded because their ages could not be determined. Of the remaining 576 voluntary donors, 10.79% tested positive for HBsAg (Table 1). Rather than expected, fewer females (12) willingly donated blood than males (572) of which 1 (8.33%) female was HBsAg + as against 62 (10.84%) males (Table 2). The age group with the highest number of donors (53.47%) was 20-29 years which also constituted the highest number of positive cases (69.35% of all positive cases) among voluntary donors (Table 3). Only 26 donors were between 40 and 60 years constituting the group with the least voluntary donors. No donor between 40 and 60 years was diagnosed HBsAg + (Table 3).

**Table 1:** Age and Gender demographics of voluntary and replacement blood donors

Age* (years)	Female (%)		Male (%)		Total (%)	
	
	VBD	RBD	VBD	RBD	VBD	RBD
11-19	10(83.33)	1(16.67)	125(22.16)	269(4.61)	135(23.44)	270(4.62)

20-29	1(8.33)	4(66.67)	307(54.43)	2,873(49.2)	308(53.47)	2,877(49.22)

30-39	1(8.33)	0(0)	106(18.79)	2,081(35.64)	107(18.58)	2,081(35.6)

40-49	0(0)	1(16.67)	26(4.61)	539(9.23)	26(4.51)	540(9.24)

50-69	-	0(0)	-	77(1.32)	-	77(1.32)

Total	12(100)	6(100)	564(100)	5,839(100)	576(100)	5,845(100)

*VBD *Voluntary blood donors; *RBD *Replacement blood donors; Pearson chi2 for VBD (3) = 24.6740; *P*-value <0.001; Pearson chi2 for RBD (4) = 4.8347 *P*-value = 0.305

**Table 2:** Cross tabulation of diagnosed status by gender of voluntary and replacement blood donors

HBV Status	Female (%)		Male (%)		Total (%)	
	
	VBD	RBD	VBD	RBD	VBD	RBD
Positive	1(8.33)	0(0)	62(10.84)	681(11.6)	63(10.79)	681(11.59)

Negative	11(91.67)	6(100)	510(89.16)	5,191(88.4)	521(89.21)	5,197(88.41)

Total	12(100)	6(100)	572(100)	5,872(100)	584(100)	5,878(100)

*VBD *Voluntary blood donors; *RBD *Replacement blood donors; Pearson chi2 for VBD (1) = 0.0767; Pr = 0.782; Pearson chi2 for RBD (1) = 0.7870 *P*-value = 0.375

**Table 3:** Cross tabulation of HBV Status of voluntary and replacement blood donors by age

Age* (years)	HBV Positive (%)		HBV Negative (%)		Total (%)	
	
	VBD	RBD	VBD	RBD	VBD	RBD
11-19	18(29.03)	38(5.6)	117(22.76)	232(4.49)	135(23.44)	270(4.62)

20-29	43(69.35)	407(60.03)	265(51.56)	2,470(47.8)	308(53.47)	2,877(49.22)

30-39	1(1.61)	190(28.02)	106(20.62)	1,891(36.6)	107(18.58)	2,081(35.6)

40-49	0(0)	40(5.9)	26(5.06)	500(9.68)	264.51)	540(9.24)

50-59	-	3(0.44)	-	74(1.43)	-	77(1.32)

Total	62(100)	678(100)	514(100)	5,167 (100)	576(100)	5,845(100)

*VBD *Voluntary blood donors; *RBD *Replacement blood donors; Pearson chi2 for VBD (3) = 18.1044; *P*-value < 0.001; Pearson chi2 for RBD (4) = 45.9008 *P*-value < 0.001

In a further analysis of a logistic regression model (Table 4), it was observed that, compared to the oldest age category for voluntary blood donors (i.e. 40-60 years), the youngest age category, 11-19 years, were 1.4 times as likely to be HBsAg+. However, the largest age category (20-29 years) were more than 2 times more likely to be HBsAg + (OR: 2.13; *p *= 0.009; 95%C.I. 1.2063-3.7488).

**Table 4:** Logistic regression of HBV status and age categories of voluntary and replacement blood donors

Age (years)	Voluntary blood donors				Replacement blood donors			
	
	OR	*p*-value	**95% C.I**.		OR	*p*-value	**95% C.I**.	
50-69	-				1.00			

40-49	1.00				1.97	0.266	0.5953	6.5411

30-39	0.06	0.006	0.0086	04604	2.48	0.126	0.7739	7.9368

20-29	2.13	0.009	1.2063	3.7488	4.06	0.018	1.2754	12.9532

11-19	1.39	0.273	0.7727	2.4938	4.04	0.023	1.2118	13.4702

*OR *Odds Ratio; *C.I*. Confidence Interval

### Replacement blood donors and HBV test results

Out of the 5,878 replacement donors including only 6 (0.1%) females, 33 recruits were excluded because their ages could not be determined (Table 1). Of the remaining 5,845 recruits, 11.59% were HBsAg+. As in volunteer blood donors, the age group with the highest number of donors (49.22%) was 20-29 years. Only 77 recruits between ages of 50 and 69 years donated blood (Table 1). Generally, male donors were also more than female donors through all age groups (Table 1).

None of the 6 female replacement donors was diagnosed with HBV. All the 11.59% of total blood replacement recruits who were positive were males (Table 2). Most positive cases (60.03%) were between 20 and 29 years followed by the 30-39 year age group with 28.02% of all positive cases. The 50-69 year age group recorded the least number of recruits likewise the least number of HBsAg + cases (Table 3).

When compared to the oldest category of replacement blood donors (50-69 years), the younger age groups 11-19 and 20-29 years were more than 4 times as likely to be HBsAg + (OR: 4.04; *p *= 0.023; 95%C.I. 1.2118-13.4702) and (OR: 4.06; *p *= 0.018; 95%C.I. 1.2754-12.9532) respectively. Those aged between 40-49 were almost twice as likely to be HBsAg+, whilst the 30-39 year old category were nearly 2½ times more likely to be HBsAg + (Table 4).

There were generally more replacement donors compared to voluntary donors. The difference in prevalence between the two groups was not significant (Chi2 = 0.6117; *p *= 0.434)

## Discussion

The liver is multifunctional. It is recognized to perform 500 functions including neutralization of toxins and drugs. This renders liver damage by HBV and other agents fatal.

### Main findings

The One Step HBsAg Test is a qualitative immunoassay that detects HBsAg in blood serum. Monoclonal and polyclonal antibodies are employed to identify HBsAg with a 1 ng/ml sensitivity. Nevertheless, the high prevalence of HBV infection (Table 1) is in concordance with some studies conducted in rural areas in developing countries where it is considered endemic [18-22]. By using a HBsAg assay with a detection limit of 1 ng/ml, the real prevalence of HBsAg carriers might even have been underestimated. Current EIA assays are showing sensitivity of <1 ng/ml. In addition, blood donors with occult HBV infection will not be detected (HBV DNA+/HBsAg-: ~1% in Ghanaian donors) [23].

Yet, the high prevalence obtained among blood donors who visited the hospital suggests that few people undergo immunization within the region. This is because the cost of immunization and/or testing is not within the means of the people. Most developing countries are facing multiple threats to the safety of blood supply because the cost of HBsAg screening is borne by the patients and their families who do not enjoy health coverage [17]. Generally also, there is no prevention of transmission between mother and child. High prevalence of HBsAg markers in donors who were >10 years is consistent with the concept that most primary HBV infections are transmitted either vertically or horizontally before age 10. Although most new infections occur among infants and young children, HBV-related morbidity and mortality is not immediately apparent [24]. The prevalence of HBsAg among women of childbearing age in Ghana is 11.5% [24]. This may also explain why low percentages of positive cases were >50 years. It is likely that by age 50 years, most carriers in the general population would have died. Low HBsAg detection in older donors might also be due to well known continuous decrease of HBsAg levels in chronic carriers over time. Most probably, HBV infection occurred before the age of 20 in these old donors. Even though there were more replacement donors, compared to voluntary donors, the difference in prevalence between the two groups was not significant (Chi2 = 0.6117; *p *= 0.434).

The reason for the gender imbalance, i.e. more males (6,444) than females (18), cannot be directly inferred from this data; however, within the context of the study area, more men than women are usually called to take responsibilities in representing families. The males are also more proactive and independent in decision making and volunteering than females would have the opportunity for. This probably explains the huge difference in gender. Illiteracy among the people in the region as well as poor access to relevant health information/education may also be a contributory factor to the high prevalence of the disease. The region is among those with the lowest school enrolment rate, highest dropout rate and highest illiteracy rates in the country. The disease is often attributed to supernatural or spiritual factors and/or alcoholism. This region is also prone to frequent ethnic conflicts which do not help the situation for intervention. Some socio cultural practices like polygamy may also enhance transmission particularly when a spouse either happens to contract the HBV elsewhere or a carrier who is apparently normal over years. The relatively high prevalence of HBV infection among the youth could be the result of risky lifestyles consistent with studies attributing it to unprotected sex [19,25-27].

## Conclusion

The prevalence of HBV infection among replacement and voluntary blood donors visiting Tamale Teaching Hospital in 2009 was estimated as 11.59% and 10.79% respectively. Both rates were high among blood donors who represent a significant part of the population within the metropolis. The probability of infection was found to be age and sex dependant. Young replacement blood donors, aged 20-29 years were 4 times as likely as older fellows (50-70 years) to be HBsAg + (OR: 4.04; *p *= 0.023, 95% C.I.:1.2118-13,4702). Among voluntary donors, the rates were lower. Compared to older donors (40-60 years) the youth group (20-29 years) were twice as likely to be HBsAg + (OR: 2.13; *p *= 0.009, 95% C.I; 1.2063-3.7488). Female donors were few to the order of 1: 358 male donors.

To reduce the aforementioned prevalence, it will be necessary for the Regional Health Directorate and NGOs to plan major HBV vaccination campaigns and educate people about risk factors for infection and benefits of immunization. It will also be necessary for the Ghana Health Service to provide kits with higher sensitivity (sensitivity of <1 ng/ml) in order to eliminate as many false positives as possible.

## Competing interests

The authors declare that they have no competing interests.

## Authors' contributions

JTD and SK conceived the study with inputs from SNI for the design of the experiments. Recruitment of blood donors and counseling was carried out by JBZ and KS. Blood sample letting and testing for HBsAg was carried out by PNA and SK, supervised by JTD. SNI led the data analysis with inputs from JTD and SK. The first draft of the paper was written by JTD, and then SK, PNA, SNI, JBZ and KS contributed to revising and reviewing the paper. All authors read and approved of the final draft before submission.
